# Clinical outcomes of COVID-19 amongst HIV patients: a systematic literature review

**DOI:** 10.4178/epih.e2021036

**Published:** 2021-05-17

**Authors:** Thomas Massarvva

**Affiliations:** Department of Primary Care and Population Health, University of Nicosia Medical School, Nicosia, Cyprus

**Keywords:** COVID-19, SARS-CoV-2, Infections, HIV, Coinfection, Acquired immunodeficiency syndrome

## Abstract

**OBJECTIVES:**

The global pandemic coronavirus disease 2019 (COVID-19) emerged in the city of Wuhan, China around December 2019. Since then, the virus has caused severe morbidity and mortality worldwide and has put pressure on the global medical system. Still, there are limited data regarding the clinical impact of COVID-19 on people living with human immunodeficiency virus (HIV). The primary aim of this study was, therefore, to systematically review up-to-date studies reporting the clinical outcomes of COVID-19 amongst HIV patients.

**METHODS:**

A thorough literature search was carried out using MEDLINE, Embase, Scopus, and the Cochrane Library Databases in accordance with the PRISMA (Preferred Reporting Items for Systematic Reviews and Meta-Analyses) guidelines.

**RESULTS:**

A total of 22 studies were identified. Amongst 730 HIV/COVID-19-coinfected patients, 79.4% were males, the median age was 51.5 years, and the number of reported patients receiving antiretroviral drugs was 708 (97.2%). Most coinfected patients had mild to moderate symptoms, including cough (37.7%), fever (37.5%), and dyspnoea (24.7%). Among pre-existing comorbidities, hypertension (26.3%) was the most prevalent in HIV/COVID-19 coinfected patients, and 87% of coinfected patients recovered.

**CONCLUSIONS:**

Based on the existing data in this systematic literature review, HIV patients with pre-existing comorbidities, obesity, and older age should be considered as a high-risk group for COVID-19. Furthermore, coinfected patients appear to have marginally comparable clinical outcomes with the general population. The study’s findings highlight the need for further investigation to elucidate the impact of COVID-19 infection on HIV patients.

## INTRODUCTION

A new pandemic, severe acute respiratory syndrome coronavirus 2 (SARS‐CoV‐2), was initially reported in Wuhan, China, on December 31, 2019 [1]. Coronavirus disease 2019 (COVID-19) is the disease caused by SARS-CoV-2, which has resulted in severe morbidity and mortality of patients worldwide. As of December 18, 2020, the World Health Organization (WHO) has reported 73,275,943 confirmed cases of COVID-19 and 1,650,348 deaths [2]. The virus spreads directly through the infected person’s droplets and body fluids, or indirectly through objects used by the infected person [3]. Its clinical characteristics range from asymptomatic to symptomatic, including respiratory disease and organ failure, leading to a substantial increase in morbidity and mortality [4].

Emerging evidence suggests that pre-existing comorbidities appear to be the driving force behind COVID-19 mortality. Amongst people with diabetes, obesity, hypertension, cardiovascular disease, respiratory diseases, stroke, dementia, chronic kidney disease, as well as old persons and immunocompromised patients, the risk of death from COVID-19 is increased [5]. Given that human immunodeficiency virus (HIV) infection results in a reduced number of CD4 cells and abnormal immune responses, leading to a weakened immune system and vulnerability to various pathogens and opportunistic infections [6], concerns about the outcomes of COVID-19 in HIV patients were immediately raised and carefully considered.

Although some scholars have speculated that antiretroviral drugs may favour HIV patients due to their activity against SARS-CoV-2 and other coronaviruses [7], there has been no evidence that HIV patients receiving certain antiretroviral drugs have an altered risk of COVID-19 infection and severity [8]. A recent study on the effectiveness of antiviral drugs has now directed focus on a drug known as tenofovir, which has been extensively used for HIV treatment and as pre-exposure prophylaxis for HIV prevention. The study found that tenofovir can bind to the RNA-dependent RNA polymerase (RdRp) of SARS-CoV-2 and may therefore impair its function [9]. While this promising finding may be useful in future research, no treatment for COVID-19 has been found. Accordingly, researchers have been trying to provide a clearer insight into various aspects of COVID-19 outcomes in HIV patients. A study found that older age, late diagnosis, low CD4 cell count, and treatment-naive status were potential determinants of COVID-19 incidence amongst HIV patients [10]. Similarly, a study in China confirmed that advanced age and preexisting comorbidities, such as hypertension and diabetes, are associated with unfavourable outcomes and increased mortality from COVID-19 [11].

Despite the current urgency to obtain a clear understanding of COVID-19 outcomes in HIV patients, large-scale observational studies on disease severity, symptoms, multimorbidity, complications, and mortality of HIV and COVID-19 coinfected patients have not yet been conducted. Given the limited data on this subject, in July 2020, the Centers for Disease Control and Prevention asserted that older HIV patients and those who have pre-existing comorbidities might be at increased risk for severe illness [12]. In view of the uncertainties relating to COVID-19 in people living with HIV and the unexpected nature of the virus, special alertness towards HIV and COVID-19 coinfection is needed. This concern is consistent with the annual rise in HIV incidence, which increases the likelihood of patients becoming coinfected with COVID-19. Despite the limited data, this systematic literature review assembled existing data on the earliest and current reported cases to provide the basis for what is known and to present relevant findings. Therefore, this study identified and quantified different aspects of COVID-19 outcomes amongst HIV patients, including the proportion of various symptoms, severity, pre-existing comorbidities, recovery, death, and the most commonly prescribed antiretroviral drugs in HIV patients with COVID-19 coinfection.

## MATERIALS AND METHODS

### Literature search

The methods and design used in this study comply with the Cochrane Collaboration Handbook, MOOSE (Meta-Analyses of Observational Studies in Epidemiology) and are reported in accordance with the PRISMA (Preferred Reporting Items for Systematic Reviews and Meta-Analyses) guidelines [13] (Supplementary Material 1). The inclusion and exclusion criteria are summarized in Table 1.

#### Information sources

A thorough literature search was carried out using the following databases: Embase, MEDLINE, Scopus, and selected Internet sites (e.g., PubMed, Cochrane Collaborative Review Group on HIV Infection and acquired immunodeficiency syndrome [AIDS], and the Science Citation Index).

#### Search strategy

Certain keywords and terms obtained from a scoping search and acquired knowledge in the field were used in the phase of literature search. Two categories of the main keywords were used for the search, COVID-19 and HIV, and related study keywords and terms were searched accordingly.

(1) COVID-19: “severe acute respiratory syndrome coronavirus” OR “coronavirus” OR “SARS-CoV-2” OR “Coronavirus Disease” OR “2019‐nCoV” OR “COVID 2019, (2) HIV: “human immunodeficiency virus” OR “Acquired Immunodeficiency Syndrome” OR “HIV” OR “AIDS” OR “HIV-1” OR “HIV-2”, (3) (1) and (2), and (4) Limit (3) to humans only.

#### Eligibility criteria

Inclusion and exclusion criteria were used for the search (Table 1). Studies that did not meet the inclusion criteria or discussed different topics that did not contribute to the research were excluded.

### Data extraction

Data were extracted using a standardized form and the assessment was carried out by classifying studies as eligible, non-eligible, or possibly eligible according to the eligibility and inclusion criteria [14]. A study was assessed for eligibility if its title and abstract were relevant and could not be excluded, and the process was based on the approval of the supervisor (Romero, R). In order to examine the data for consistency and clarity or in case of disagreement on the inclusion of studies, a third reviewer could be appointed to assess eligibility independently. The following data were extracted: sample size, study design, population characteristics (age, sex, CD4 cell count, viral load), used antiretroviral drugs, outcome (recovery, death, still in hospital), severity (mild, moderate, severe/critical) and related comorbidities (e.g., diabetes mellitus, hypertension, cardiovascular diseases, respiratory diseases, chronic kidney disease, obesity). Where necessary, authors were asked to provide the raw data or additional information.

### Quality assessment

The quality and risk of bias assessment was carried out using the Newcastle-Ottawa Quality Scale [15], which includes 8 customized assessment sheet criteria that are categorised into 3 groups: selection, comparability, and outcome. The assessment sheet evaluated the representativeness of cases, research methods and the outcomes of each study. Quality assessment was conducted using different criteria based on various study designs. A score between 1 and 3 out of 8 implies a higher risk of bias due to inadequacy of reporting, the use of invalid and unreliable measures to define conditions and exposures, mis-representativeness of cases, unclear population characteristics, unblinded assessors, and insufficient follow-up of cases. Medium-risk studies scored 4-5 out of 8 and lower-risk studies scored 6-8 out of 8 (Supplementary Material 2).

### Data synthesis

Data from the included studies were tabulated and categorized based on the study sample, comorbidities, recovery, severity, antiretroviral drug use, and other factors influencing the total number of patients. Accordingly, a descriptive analysis was used to report findings, and bar charts were used to display key findings. Stata version 16.0 (Stata Corp., College Station, TX, USA) was used for the analysis.

Unfortunately, due to a lack of data and variability in the comparison groups amongst the included studies, a meta-analysis for comparison between groups could not be performed. However, when data permitted, a meta-analysis of proportions was conducted by calculating the pooled estimate of the proportion of recovery amongst patients with HIV/COVID-19 coinfection, using a random-effects model [16]. The meta-analysis of proportions was performed using the “metafor” package in R version 4.0.3 (R Foundation for Statistical Computing, Vienna, Austria). Data were weighted prior to combination to account for the various samples from different studies and to avoid pooling the data as if they were derived from a single sample.

A random-effects analysis was used, with a binomial distribution modelling within-study variability and parameters estimated using a maximum likelihood procedure. To calculate the confidence intervals (CIs) for between-study variance, the Jackson [17] method was used. The overall proportions were shown, along with 95% CIs. The I^2^ statistic was used to report between-study heterogeneity, and the p-value confirmed the result.

### Publication bias assessment

Publication bias was assessed using funnel plots, and the degree of asymmetry was tested using Egger’s regression test [18]. Both analyses were performed in R using the “metafor” package.

### Ethics statement

As the present study was a systematic review, no ethics statement was needed.

## RESULTS

### Study selection

A total of 632 articles were found. Following the PRISMA guidelines, 247 duplicate articles were excluded and after screening titles and abstracts, 339 additional articles were excluded. Only 22 of the studies were included for analysis after full-text screening of 44 articles.

**Figure 1.**
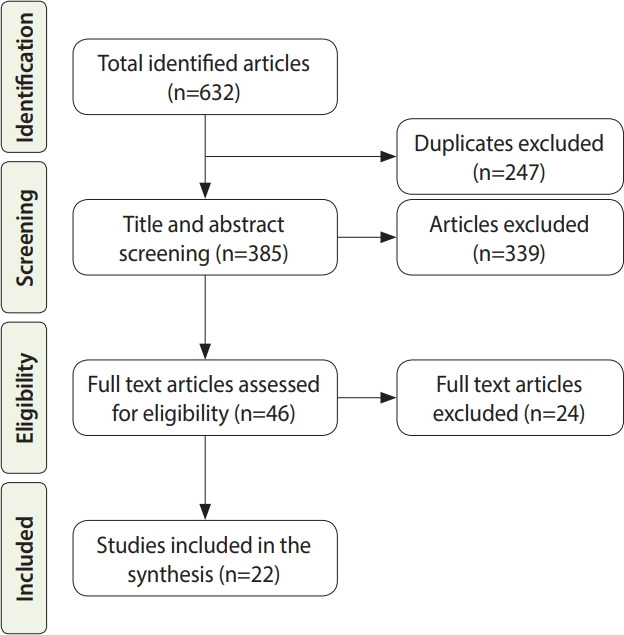
PRISMA (Preferred Reporting Items for Systematic Reviews and Meta-analysis) flow chart of search and study selection.

All 22 included articles were observational studies, of which there were 9 cohort studies (including 3 retrospective studies), 9 case series, 2 cross-sectional studies, 1 case-control study, and 1 case report. Figure 1 shows a PRISMA flow chart that explains the inclusion and exclusion process for this systematic literature review.

### Study characteristics

The finally selected studies were listed according to the first author, publication year, country, study design, sample, antiretroviral drugs, findings, and outcomes. The study characteristics are detailed in Table 2 [10,19-39]. All of the selected studies were observational studies that provided data on the outcomes of COVID-19 amongst HIV patients, clinical outcomes amongst antiretroviral therapy (ART) users, demographic data, and severity determinants in coinfected cases. Six studies were conducted in the United States, 6 in China, 3 in Spain, 2 in Italy, and the remaining 5 studies were conducted in France, Germany, United Kingdom, Turkey, and Iran. All of the studies were conducted between April 15, 2020 and November 17, 2020. An overview of the included studies is provided in Table 3 [10,19-39].

### Quality and risk of bias within studies

The quality of studies and risk of bias were reported using the Newcastle-Ottawa Scale following approval from the supervisor (Romero, R). Although all of the included studies are observational, different criteria were customised to assess the quality and bias within studies based on the study design. The assessment was carried out using a rating scale ranging from 1 to 8 out of 8. A higher score implies a lower risk of bias due to adequacy of reporting, the use of valid and reliable measures to define conditions and exposures, representativeness of cases, presence of nonexposed in cohorts, clarity of characteristics, blinding assessors, sufficient follow-up of cases, and clear outcomes. None of the included studies scored lower than 4, and therefore none were classified as high-risk studies. Where certain data were missing and no additional information could be received from the authors, the ratings were classified as “unknown” (Supplementary Material 2).

### Synthesis of results

The synthesis of results from different observational study designs across outcomes of COVID-19 amongst HIV patients is provided in Table 4. Overall, 730 HIV patients with COVID-19 coinfection were reported in all studies. The patients were predominantly male (79.4%) and the mean age of all reported cases was 49.11 years. A low CD4 cell count (< 200 cells/mm^3^) was reported in 87 of 470 patients (18.5%) and the viral load was higher than 50 copies/mL in 41 of 393 patients (10.4%). The number of reported patients receiving antiretroviral drugs was 708, representing 97.2% of all cases, and only 22 patients were treatment-naive.

**Figure 2.**
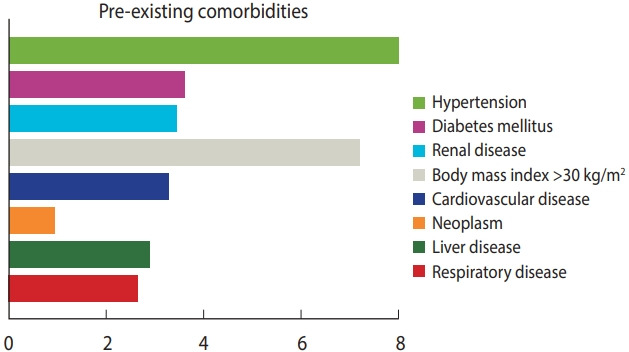
Mean proportion of patients with different comorbidities in the included studies.

**Figure 3.**
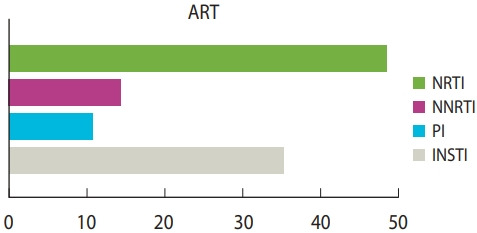
Mean proportion of patients receiving different antiretroviral medications in the included studies. ART, antiretroviral therapy; NRTI, nucleoside reverse transcriptase inhibitor; NNRTI, non-nucleoside reverse transcriptase inhibitor; PI, protease inhibitor; INSTI, integrase strand transfer inhibitor.

While 81.9% of coinfected patients had mild to moderate symptoms of COVID-19, severe symptoms were diagnosed in 18.1% of the cases. The pre-existing comorbidities associated with HIV/COVID-19-coinfected patients were hypertension (26.3%, 128 patients, 16 studies), body mass index greater than 30 kg/m2 (14.8%, 72 patients, 10 studies), diabetes mellitus (12.5%, 61 patients), renal diseases (12.1%), cardiovascular diseases (11.5%) and liver disease (10.1%) (Figure 2 and Table 4). Only 11 studies reported symptoms of COVID-19 amongst HIV patients. The most common symptoms were cough (37.7%), fever (37.5%), and dyspnoea (24.7%). Of the 720 reported cases, 90.6% recovered and 9.4% (n= 68) died. The antiretroviral regimens were grouped into 4 subcategories (nucleoside reverse transcriptase inhibitors [NRTIs], non-nucleoside reverse transcriptase inhibitors [NNRTIs], protease inhibitors [PI], and integrase strand transfer inhibitors [INSTIs]). At the time of diagnosis, a significantly higher proportion of individuals with COVID-19 (44.6%) received a tenofovir-based regimen (NRTI), while 32.4% received INSTI (Figure 3 and Table 4).

**Figure 4.**
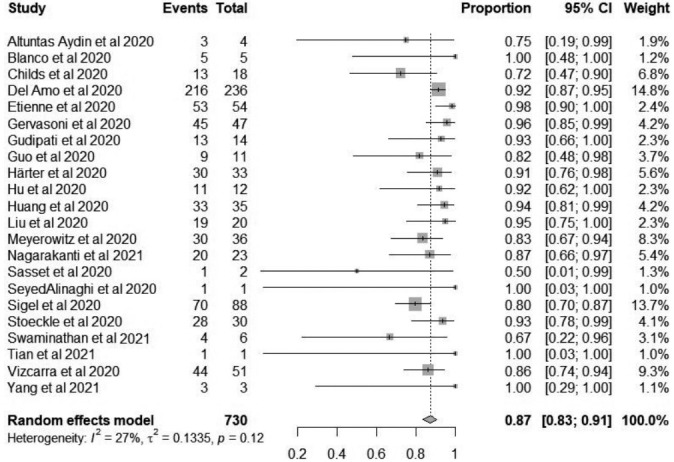
Forest plot for the recovery proportion of coronavirus disease 2019/human immunodeficiency virus-coinfected patients amongst the included studies. CI, confidence interval.

When data allowed, the meta-analysis of proportions included all 22 studies to estimate the recovery proportion of HIV/COVID-19-coinfected patients. None of the included studies were excluded from the analysis due to the low-medium risk of bias (scored 4-8). The pooled recovery proportion of HIV/COVID-19 coinfection was 87% (95% CI, 83 to 91), as shown in Figure 4. The weighted mean of the estimates from each included study revealed differences in sample sizes between studies. However, using a random-effects model, the results showed an insignificant degree of heterogeneity between studies, as confirmed by the p-value (I^2^= 27%, p= 0.12, π^2^= 0.1335). Although low, a considerable amount of the variability between studies (I^2^= 27%) could be attributed to clinical and methodological differences; for example, some studies included patients with multiple morbidities and those of older age, both of which are risk factors for a severe course of COVID-19, resulting in a lower recovery ratio. Furthermore, this systematic literature review included various observational study designs that may have contributed to a relatively high degree of variability.

### Publication bias

The funnel plot showed a symmetrical scattering of points throughout the funnel, indicating that publication bias was unlikely. Furthermore, the Egger regression test revealed no significance in the asymmetry degree of the funnel plot (p= 0.85) (Supplementary Material 3).

## DISCUSSION

By December 2020, more than 73 million confirmed cases of COVID-19 and more than 1.6 million worldwide deaths had been reported by the WHO [2]. In line with the aim of this study, 730 patients coinfected with COVID-19 and HIV were investigated. The majority of patients were male and most of them received antiretroviral drugs. Furthermore, 81.9% of coinfected cases had mild to moderate clinical symptoms, primarily including cough, fever, and dyspnoea [20,35]. Due to the impaired immune defences due to the underlying disease, including in patients receiving treatment, HIV patients were thought to be at higher risk of developing severe forms of COVID-19 [40]. Although comparative tests were not possible due to the limited data and differences in comparative groups between studies, some of the included studies revealed a similar representation of people living with HIV across the population. An earlier cohort from New York City, United States found no differences in adverse outcomes between HIV/COVID-19-coinfected patients and a similar comparison group [34]. In Wuhan, China, a cohort study indicated that case-severity rates of COVID-19 in HIV patients were comparable to those in the overall population [28], and in Italy, coinfected HIV patients with COVID-19 were found to be at no higher risk of severe illness than HIV-negative patients [24].

While further research is needed to provide evidence regarding the prognosis of COVID-19 in HIV patients and other groups, this study has shown that older age and the presence of comorbidities, including hypertension, obesity, diabetes mellitus, renal diseases, cardiovascular diseases, chronic respiratory disease, liver disease, and malignancy, are associated with a poor prognosis in COVID-19 patients, including the risk of death. This aligns with an earlier report finding that the most prevalent comorbidities among COVID-19 patients were hypertension, obesity, chronic lung disease, and diabetes mellitus [41]. Further evidence on the relationship between pre-existing comorbidities and adverse outcomes of COVID-19 was reported in a previous systematic review, which found that older age and the presence of one or more comorbidities increased COVID-19 severity [42], and these findings also align with those presented by Meyerowitz et al. [30] who reported that 83% of all HIV/COVID-19-coinfected patients had comorbidities associated with severe COVID-19 outcomes. Considering the age factor, a relatively large cohort study found that older patients (median age, 63 years) had a higher risk of developing severe disease [43]. Despite the lower median age in this systematic literature review (51.5 years), some of the included studies confirmed a similar association in coinfected patients with COVID-19 and HIV [23,29], taking into account that the median age difference may be due to the changing age distribution of COVID-19 between May 2020 and August 2020, which is thought to be due to behavioural and occupational factors as younger people are less likely to follow safety precautions [44]. A similar age shift was identified in Europe, where the median age of COVID-19 cases was 54 years in the first half of 2020 and then decreased to 39 years during June-July 2020 [45].

As of December 31, 2020, almost 68 million (97%) people had recovered from COVID-19 infection worldwide [2]. Despite the high recovery percentage globally, this systematic literature review showed a lower percentage of recovery in both a simple pooling of unweighted studies (90.6% recovery) and a meta-analysis of proportions following weighting of studies (87.0% recovery). These findings are consistent with the high proportion of death amongst HIV/COVID-19-coinfected patients (9.4%). However, different studies have shown conflicting findings regarding the coinfection-related deaths in patients with HIV and COVID-19. Huang et al. [28] reported a lower percentage of death related to coinfection (5.71%). A study in France [23], in contrast, reported a considerably lower percentage of deaths (1.8%). This significant variation of results is most likely due to selection bias, as many patients were admitted to hospital with severe symptoms. Another reason may be the presence of confounders, including certain comorbidities or determinants that increase the risk of death from COVID-19, such as hypertension, obesity, diabetes mellitus, and old age [42], which is consistent with the findings of the included studies in this systematic literature review [19,23,25,26,34,36].

Owing to the increase in the number of COVID-19 cases worldwide and the global demand for treatment, some HIV antiviral drugs have been under the spotlight. This particular interest was driven by a finding from the 2004 severe acute respiratory syndrome (SARS) outbreak, where it was observed that none of 19 HIV/AIDS patients receiving ART had contracted SARS despite being in close contact with SARS patients [46], which led to the hypothesis that the use of ART could prevent SARS from developing and could potentially reduce the severity and mortality of COVID-19. While the previous literature did not provide conclusive evidence on the effect of ART against SARS-CoV-2 infection [47], Elfiky [9] found that various antiviral drugs, including ribavirin, remdesivir, sofosbuvir, galidesivir, and tenofovir (NRTI) showed potential activity against SARS-CoV-2 by binding to its RdRp. This finding is congruous with previous studies suggesting that NRTIs inhibit the RdRp of COVID-19 and therefore may be effective against COVID-19 infection [48,49]. While this systematic literature review found that NRTIs were the most commonly used type of ART amongst HIV/COVID-19 patients, this finding cannot explain the relatively low severity of COVID-19 amongst HIV patients. Future research is therefore needed to further understand the role of antiretroviral drugs in reducing the severity of COVID-19 amongst HIV patients.

### Strengths and limitations

In view of the rapidly changing evidence of the ongoing global pandemic, this study has been continuously updated over the course of 2020 and therefore includes relevant data. This research addresses an important and interesting concern that adds to the essential information needed to further understand the impact of COVID-19 on HIV patients. Considering the unstable and mutable nature of COVID-19, this study provides an up-to-date and in-depth analysis of the clinical outcomes of COVID-19 amongst HIV patients. Furthermore, the results highlight additional evidence on the clinical determinants of COVID-19 severity in HIV patients and their impact on patients’ outcomes and quality of life. There are also some limitations to this study. First, most of the included studies have coinfected patients with a CD4 cell count greater than 200 cells/mm^3^ and an HIV viral load lower than 50 copies/mL; thus, the results may not represent uncontrolled HIV patients. Second, while data on the antiretroviral drugs used in coinfected patients with HIV/COVID-19 were available, a comparison between the antiretroviral drugs received in coinfected cases of HIV/COVID-19 and HIV without COVID-19 was not possible due to lack of information. Some of the included studies lacked a comparison group and therefore provided limited information on the determinants associated with HIV/COVID-19 coinfection. For some of the results, the findings were influenced by a single large study [22]. Furthermore, most of the included studies had small samples, and various confounders were not included in the reporting of such data.

## CONCLUSION

Based on the findings of this systematic literature review, coinfected patients with HIV/COVID-19 have marginally comparable clinical outcomes with the general population. HIV patients with pre-existing comorbidities should be considered as a high-risk COVID-19 group, along with those who are obese and older. Healthcare providers will therefore need to take particular care of HIV patients with multiple morbidities to prevent unnecessary poor outcomes. Given the mutable nature of COVID-19, HIV patients should still be advised to take additional precautions and protect themselves as new information is rapidly updated. Despite the lack of evidence on the effectiveness of antiretroviral drugs against COVID-19, controlled HIV patients with an undetectable HIV viral load appear to have better outcomes than uncontrolled patients. HIV patients should therefore be encouraged to adhere to their therapy. These findings highlight the need for further investigation of the impact of COVID-19 infection on HIV patients.

## Figures and Tables

**Table 1.** Inclusion and exclusion criteria for the search

Characteristics	Inclusion criteria	Exclusion criteria
Diagnosis	Confirmed or probable diagnosis of COVID-19 amongst HIV patients.	Studies discussing environmental, economic, or social impacts of COVID‐19 on HIV patients
Age (yr)	>16	<16
Type of study	Studies in English language and no geographical restrictions	Pre-clinical studies and news reports, editorials, reviews
Published date	Between Apr and Dec 2020	Before Apr 2020

COVID-19, coronavirus disease 2019; HIV, human immuno deficiency virus.

**Table 2.** Characteristics of the included studies

Study, country	Study design	Sample	Antiretroviral drugs	Findings and outcomes
Altuntas Aydin et al., 2020 [19]	Case series	4 HIV patients with confirmed COVID‐19	3 of the 4 patients were on ART; all three patients received NRTI (tenofovir-based regimen/emtricitabine) and INSTI (dolutegravir/ elvitegravir)	- Use of regular antiretroviral drugs and suppression of viral load improved COVID-19 outcomes amongst HIV patients
Turkey				- Pre-existing comorbidities were an important factor in the mortality of COVID-19/HIV-coinfected patients
				- 1 death occurred due to untreated comorbidities
Blanco et al., 2020 [20]	Case series	Of 543 confirmed COVID-19 cases, 5 patients were HIV-positive	4 patients were on ART; 2 patients received PI (darunavir-boosted cobicistat), and 2 patients received INSTI (dolutegravir)	- COVID-19/HIV-coinfected patients had similar symptoms of COVID-19 as the general population
Spain				- 4 patients were cured and 1 remained in the ICU at the end of the study
Childs et al., 2020 [21]	Case series	18 COVID-19/HIV-coinfected patients	All 18 patients were on ART; all of them received NRTI, 11 received PI, 4 received NNRTI, and 3 received INSTI	- Hospitalized patients with COVID-19 were more likely to be of Black ethnicity and to have a lower CD4 cell count
United Kingdom				- 5 patients died, 12 patients recovered, and 1 patient remained in the hospital at the end of the study
Del Amo et al., 2020 [22]	Cohort study	236 COVID-19/HIV-coinfected patients out of 77,590 HIV patients receiving ART	All COVID-19/HIV-coinfected patients received NRTIs; 21 received TDF/FTC (tenofovir disoproxil fumarate/emtricitabine, 100 received TAF/FTC (tenofovir alafenamide/ emtricitabine), and 57 received ABC/3TC	- 151 coinfected patients with COVID-19/HIV were hospitalized and 15 were admitted to the ICU
Spain				- The risk of diagnosis of COVID-19 was lower in HIV patients (30.0 per 10,000) than in the general population (41.7 per 10,000)
				- This risk of diagnosis of COVID-19 was lower among HIV patients who received TDF/FTC
				- 20 patients died, of whom 10 were on TAF/FTC, 8 were on ABC/3TC, and none were on TDF/FTC
Etienne et al., 2020 [23]	Cohort study	54 COVID-19/HIV-coinfected patients	All 54 patients were on ART; 43 patients received NRTI, 33 received INSTI, and 25 received NNRTI	- COVID-19 severity in HIV patients was associated with male sex, older age, and metabolic disorders including diabetes mellitus and obesity
France				- 1 patient died
Gervasoni et al., 2020 [24]	Case series	Of 6,000 HIV patients, 47 had proven or probable COVID-19 infection	80% of COVID-19/HIV-coinfected patients received INSTI, 11% received PI, and 42% received NRTI	- 13 COVID-19/HIV-coinfected patients were hospitalized
Italy				- The risk of severe disease, death, and admission to an ICU in COVID-19/HIV-coinfected patients compared favourably with that seen in the entire population of COVID-19 patients
				- 2 deaths occurred
Gudipati et al., 2020 [25]	Case series	14 HIV patients had confirmed COVID-19 infection from 7,372 COVID-19 positive tests	13 of the 14 coinfected patients received ART; 12 patients received tenofovir-based regimen (NRTI); one patient received a PI-based regimen	- 8 patients were hospitalized, and 2 patients were transferred to the ICU (both of whom have pre-existing comorbidities)
USA				- 1 patient died as a result of cardiac arrest
				- Coinfected patients with COVID-19/HIV were not at a higher risk of death or severe outcomes than HIV-negative patients
Guo et al., 2020 [10]	Cross-sectional study	11 HIV/COVID-19-coinfected patients	10 HIV patients with COVID-19 were on ART therapy; 9 patients received NRTI and NNRTIs, 1 patient received lopinavir/ritonavir (PI)	- The incidence rate of COVID-19 in HIV patients in Wuhan, China was comparable to that of the entire population (0.6%)
China				- Those who were of older age and treatment-naïve showed a marginal association with contracting COVID-19
				- 2 deaths
Härter et al., 2020 [26]	Case series	33 COIVID-19/HIV-coinfected patients	All patients were on ART; 31/33 received NRTIs, 20 received INSTI and 9 received NNRTI	- 91% of all COVID-19/HIV patients recovered and 76% were classified as mild cases
Germany				- 3/32 patients died (9.3%), of whom 1 patient was 82 yr old, the second patient had a CD4 cell count below 200/mm^3^, and the third patient suffered from several comorbidities
Hu et al., 2020 [27]	Case series	Of 2,900 HIV patients, 12 COVID-19/HIV-coinfected patients	9 COVID-19/HIV patients received ART; all of them received NRTI and NNRTI	- Late initiation of ART among HIV patients could lead to more severe symptoms
China				- 1 patient died
Huang et al., 2020 [28]	Cohort study	35 of 6,001 (0.6%) HIV patients had COVID-19	28/35 (80.0%) were on continued ART; 4 patients (11.4%) had discontinued their ART therapy; 32/35 (91.4%) received NRTI; 30/35 (80.5%) received NNRTI	- The incidence, case-fatality, and severity rates of COVID-19 in HIV patients were comparable to those in the entire population
China				- COVID-19 incidence amongst HIV patients on ART was lower than those who had discontinued therapy or were treatment-naive
				- The COVID-19 incidence rate amongst HIV patients aged 50 yr or above was 3 times higher than amongst HIV patients younger than 50 yr
				- 2/35 deaths of patients coinfected with COVID-19/HIV in compared to 3,869/50,333 in the entire population
Liu et al., 2020 [29]	Retrospective cohort study	20 COVID-19/HIV-coinfected patients	12 of 20 patients were on ART and 8 patients were treatment-naive; all 12 patients received NRTIs; 8 of them were on PIs, mainly lopinavir/ritonavir, and 6 were on NNRTI (efavirenz)	- Most COVID-19/HIV-coinfected patients (85%) presented with mild to moderate symptoms, which may be associated with ART history in HIV patients
China				- 1 death: an old man with pre-existing comorbidities
Meyerowitz et al., 2020 [30]	Cohort-based study	36 patients living with HIV were diagnosed with COVID-19	35 patients were on ART; 29/35 received INSTI; 9 received NNRTI, 4 received PI, and 30 received NRTI	- 58% of COVID-19/HIV-coinfected patients including 8 severe and 7 critical cases required hospitalization
USA				- 30 patients (83.3%) had severe illness associated with pre-existing comorbidities
				- 2 patients (5.6%) died
Nagarakanti et al., 2021 [31]	Retrospective cohort study	23 COVID-19/HIV-coinfected patients	35% of patients received INSTI, 22% received NNRTI, and 26% received a combination of INSTI and PI	- Clinical outcomes were similar between COVID-19/HIV-coinfected patients and COVID-19 patients without HIV
USA				- 3 patients died
Sasset et al., 2020 [32]	Case series	2 COVID-19/HIV-coinfected patients	Both patients received INSTI and PI	- Both patients suffered from several comorbidities
Italy				- 1 patient recovered and the second was still in the ICU at the end of this report
				- Only 0.15% of the total HIV population showed COVID-19 symptoms
SeyedAlinaghi et al., 2020 [33]	Cross-sectional study	Of 200 HIV patients, one patient had COVID-19	The patient received lopinavir/ritonavir (PI) and Truvada (NRTI)	- Symptoms resolved in 1 wk
Iran				- HIV-positive patients and/or patients receiving ART may have a lower susceptibility to becoming infected with COVID-19 or have a decreased severity of the disease
Sigel et al., 2020 [34]	Cohort study	Of 439 COVID-19 patients, 88 were coinfected with HIV	All 88 coinfected patients were on ART; 85 patients received NRTI, 69 received INSTI, and 15 received PI	- During the follow-up period, most COVID-19/ HIV patients were discharged from the hospital
USA				- 18 patients died, most of whom had comorbidities such diabetes, hypertension, and chronic kidney disease
Stoeckle et al., 2020 [35]	Retrospective cohort study	30 COVID-19/HIV-coinfected patients (case group) matched with 90 COVID-19 patients without HIV (control group)	29/30 patients were on ART; 9 patients received PI (lopinavir/ritonavir) and 19 received NRTI	- Symptoms and laboratory findings were similar between cases and controls
USA				- Immunity suppression in HIV patients may result in less severe forms of COVID-19 and potentially favourable outcomes
				- No deaths
Swaminathan et al., 2021 [36]	Case series	6 COVID-19/HIV-coinfected patients	5/6 patients received ART; the majority of the patients were on INSTIs	- Some data suggests that the protective effect of antiretroviral drugs could result in a favourable outcome of COVID-19 in HIV patients
USA				- 2 deaths (both had multiple medical comorbidities)
Tian et al., 2021 [37]	Case report	1 HIV/COVID-19-coinfected patient	The patient received lopinavir/ritonavir (PI)	- HIV patients who received regular antiretroviral drugs had no severe outcomes or a poor prognosis of COVID-19
China				- No deaths
Vizcarra et al., 2020 [38]	Cohort study	51 of 1,339 HIV patients were diagnosed with COVID-19	37 (73%) COVID-19/HIV-coinfected patients received NRTI and 41(80%) received INSTI	- Of all coinfected cases, 44 patients recovered
Spain				- Previous use of NRTIs or PIs was not associated with differences in the clinical presentation
				- 2 deaths
Yang et al., 2021 [39]	Case-control study	3 COVID-19/HIV-coinfected patients (case group)	All coinfection patients received ART; 2 patients received NRTI and NNRTI; the third patient received NRTI INSTI, NNRTI	- The effects of antiretroviral drugs in the prevention and treatment of COVID-19 may be favourable, but seem to be limited
China		53 COVID-19 patients without HIV (control group)		- No deaths

COVID-19, coronavirus disease 2019; HIV, human immunodeficiency virus; ART, antiretroviral therapy; NRTI, nucleoside reverse transcriptase inhibitor; NNRTI, non-nucleoside reverse transcriptase inhibitor; PI, protease inhibitor; INSTI, integrase strand transfer inhibitor; ICU, intensive care unit; TDF, tenofovir disoproxil fumarate; FTC, emtricitabine; TAF, tenofovir alafenamide; ABC, abacavir; 3TC, lamivudine.

**Table 3.** Overview of the population characteristics from observational studies

Study	Sample (COVID-19/HIV-coinfected patients), n	Age (mean), yr	Sex, n	CD4 cell count, (>200 cells/ mm^3^) n	HIV viral load, (<50 copies/ mL) n	Severe/critical cases, n	Comorbidities	Symptoms	Antiretroviral drugs
Altuntas Aydin et al., 2020 [19]	4	37.2	M: 4	3	3	1	HTN, DM, BMI>30 kg/m^2^	Fever, cough, dyspnoea	NRTI, INSTI
Blanco et al., 2020 [20]	5	37.8	M: 3, F: 2	5	4	2	Hypothyroidism, asthma	Fever, cough, dyspnoea	PI, INSTI
Childs et al., 2020 [21]	18	52.0	M: 12, F: 6	18	17	5	HTN, DM, RD, BMI>30 kg/m^2^	Fever, cough, dyspnoea	NRTI, NNRTI, PI, INSTI
Del Amo et al., 2020 [22]	236	47.0	M: 204, F: 32	U	U	15	U	U	NRTI, NNRTI, PI, INSTI
Etienne et al., 2020 [23]	54	54.0	M: 33, F: 21	51	51	19	HTN, DM, RD, CVD, RPD, LD, neoplasm, BMI>30 kg/m^2^	U	NRTI, NNRTI, PI, INSTI
Gervasoni et al., 2020 [24]	47	51.0	M: 36, F: 11	36	44	6	HTN, DM, RD, CVD, LD, RPD, neoplasm	Fever, cough, dyspnoea	NRTI, PI, INSTI
Gudipati et al., 2020 [25]	14	49.0	M: 12, F: 2	12	13	2	HTN, DM, RD, CVD, RPD, BMI>30 kg/m^2^	Fever, cough, dyspnoea	NRTI, PI
Guo et al., 2020 [10]	11	53.2	M: 10, F: 1	9	9	5	6 cases with comorbidities	U	NRTI, NNRTI, PI
Härter et al., 2020 [26]	33	48.0	M: 30, F: 3	31	30	8	HTN, DM, RD, CVD, RPD, LD	Fever, cough, dyspnoea	NRTI, NNRTI, PI, INSTI
Hu et al., 2020 [27]	12	36.0	M: 10, F: 2	12	8	2	HTN, RD, RPD	U	NRTI, NNRTI
Huang et al., 2020 [28]	35	52.0	M; 33, F: 2	30	22	15	U	U	NRTI, NNRTI
Liu et al., 2020 [29]	20	46.5	M: 5, F: 15	U	U	3	HTN, DM, CVD, RPD, LD	Fever, cough, dyspnoea	NRTI, NNRTI, PI
Meyerowitz et al., 2020 [30]	36	53.4	U	34	U	15	HTN, DM, RD, BMI>30 kg/m^2^	U	NRTI, NNRTI, PI, INSTI
Nagarakanti et al., 2021 [31]	23	59.0	M: 14, F: 9	20	U	2	HTN, DM, RD, CVD, RPD	Fever, cough, dyspnoea	NRTI, NNRTI, PI, INSTI
SeyedAlinaghi et al., 2020 [33]	1	40.5	M: 1	N	1	N	N	Fever, cough, dyspnoea	NRTI, PI
Sigel et al., 2020 [34]	88	61.0	M: 66, F: 22	64	66	18	HTN, DM, RD, LD, RPD, neoplasm, BMI>30 kg/m^2^	U	NRTI, NNRTI, PI, INSTI
Stoeckle et al., 2020 [35]	30	60.5	M: 24, F: 6	20	27	4	HTN, DM, RD, CVD, LD, RPD,	Fever, cough, dyspnoea	NRTI, PI
Sasset et al., 2020 [32]	2	61.2	M: 2	2	2	2	HTN, CVD, LD, BMI>30 kg/m^2^	U	PI
Swaminathan et al., 2021 [36]	6	64.0	M: 5, F: 1	6	5	2	HTN, DM, RD, CVD, RPD, BMI>30 kg/m^2^	U	INSTI
Tian et al., 2021 [37]	1	24.0	M: 1	1	U	N	U	U	PI
Vizcarra et al., 2020 [38]	51	53.3	M: 43, F: 8	27	50	6	HTN, DM, RD, CVD, LD, RPD, neoplasm, BMI>30 kg/m^2^	Fever, cough, dyspnoea	NRTI, NNRTI, PI, INSTI
Yang et al., 2021 [39]	3	40.0	M: 3	2	U	N	U	U	NRTI, NNRTI, INSTI

U, unknown; N, null; HTN, hypertension; DM, diabetes mellitus; RD, renal disease; CVD, cardiovascular disease; BMI, body mass index; LD, liver disease; RPD, respiratory disease; NRTI, nucleoside reverse transcriptase inhibitor; NNRTI, non-nucleoside reverse transcriptase inhibitor; PI, protease inhibitor; INSTI, integrase strand transfer inhibitor; M, male; F, female.

**Table 4.** Demographic and clinical characteristics of COVID-19 infection in people living with HIV included in the reviewed studies (total patients=730)

Characteristics	N (%)
Age (mean±SD/median)	49.11±9.94/51.5
Sex (n=694)	
Male	551 (79.4)
Female	143 (20.6)
Used antiretroviral drugs (n=728)	
Yes	708 (97.2)
No	22 (2.7)
CD4 cell count (n=470)	
<200/mm^3^ (ns=20)	87 (18.5)
≥200/mm^3^ (ns=20)	383 (81.5)
HIV viral load (n=393)	
<50 copies/mL (ns=16)	352 (89.6)
>50 copies/mL (ns=15)	41 (10.4)
Severity (n=728)	
Mild-moderate	596 (81.9)
Severe-critical	132 (18.1)
Clinical outcome (n=720)	
Death	68 (9.4)
Recovery	652 (90.6)
ART regimen	
NRTI	543 (44.6)
NNRTI	161 (13.2)
PI	119 (9.8)
INSTI	395 (32.4)
Comorbidities	
Hypertension (ns=16)	128 (26.3)
Body mass index >30 kg/m^2^ (ns=10)	72 (14.8)
Diabetes mellitus (ns=17)	61 (12.5)
Renal disease/CKD/ESRD (ns=17)	59 (12.1)
Cardiovascular disease (ns=17)	56 (11.5)
Neoplasm (ns=17)	16 (3.3)
Liver disease (ns=17)	49 (10.1)
Respiratory disease (ns=17)	45 (9.3)
Symptoms (n= 453)	
Cough (ns=11)	171 (37.7)
Fever (ns=11)	170 (37.5)
Dyspnoea (ns=11)	112 (24.7)

COVID-19, coronavirus disease 2019; HIV, human immunodeficiency virus; N, number of the variable; n, number of reported cases; ns, number of analysed studies; SD, standard deviation; ART, antiretroviral therapy; NRTI, nucleoside reverse transcriptase inhibitor; NNRTI, non-nucleoside reverse transcriptase inhibitor; PI, protease inhibitor; INSTI, integrase strand transfer inhibitor; CKD, chronic kidney disease; ESRD, end-stage renal disease.

## SUPPLEMENTARY MATERIALS

Supplementary Material 1. PRISMA checklist

Supplementary Material 2. The Newcastle Ottawa scale for quality assessment of observational studies

Supplementary Material 3. Funnel plot for the assessment of publication bias following a meta-analysis of proportion.
