# Supplementary material for: Clinical outcomes of COVID-19 amongst HIV patients: a systematic literature review
Source: Epidemiol Health. 2021 May 17;43:e2021036. doi: 10.4178/epih.e2021036 (PMC8342867; doi:10.4178/epih.e2021036)
Supplement: Supplementary Material 2. — The Newcastle Ottawa scale for quality assessment of observational studies [file epih-43-e2021036-suppl2.docx]

|  | **The Newcastle Ottawa scale for quality assessment of cohort studies (Prospective& Retrospective)** |  |  |  |  |  |  |  |  |  |
| --- | --- | --- | --- | --- | --- | --- | --- | --- | --- | --- |
|  |  | Authors |  |  |  |  |  |  |  |  |
|  | Category | Del Amo et al, 2020 | Etienne et al, 2020 | Huang et al, 2020 | Keith et al, 2020 | Liu et al, 2020 | Meyerowitz et al, 2020 | Nagarakanti et al, 2020 | Stoeckle et al, 2020 | Vizcarra et al, 2020 |
| A | Selection |  |  |  |  |  |  |  |  |  |
|  | Representativeness of exposed cohort | 🗸 | 🗸 | 🗸 | 🗸 | × | 🗸 | 🗸 | × | 🗸 |
|  | Selection of the non-exposed from same community | 🗸 | 🗸 | 🗸 | 🗸 | 🗸 | × | 🗸 | 🗸 | 🗸 |
|  | Ascertainment of exposure by Secured records or structured interview | 🗸 | 🗸 | 🗸 | 🗸 | 🗸 | 🗸 | 🗸 | 🗸 | 🗸 |
|  | Demonstration that outcome of interest was not present at start of study | 🗸 | 🗸 | 🗸 | 🗸 | × | × | × | × | 🗸 |
|  |  |  |  |  |  |  |  |  |  |  |
| B | Comparability |  |  |  |  |  |  |  |  |  |
|  | Study controls for other variables | 🗸 | 🗸 | 🗸 | 🗸 | 🗸 | × | 🗸 | 🗸 | 🗸 |
|  |  |  |  |  |  |  |  |  |  |  |
| C | Outcome |  |  |  |  |  |  |  |  |  |
|  | Assessment of outcome (Independent blind assessment, secured record)? | 🗸 | × | 🗸 | 🗸 | 🗸 | 🗸 | 🗸 | 🗸 | 🗸 |
|  | Was follow-up long enough for outcome to occur | 🗸 | 🗸 | 🗸 | U | U | U | U | U | 🗸 |
|  | Adequacy of follow-up of cohorts (complete follow-up)? | 🗸 | 🗸 | 🗸 | 🗸 | 🗸 | 🗸 | 🗸 | 🗸 | 🗸 |
|  |  |  |  |  |  |  |  |  |  |  |
|  | Score | 8 | 7 | 8 | 7 | 5 | 4 | 6 | 5 | 8 |

**U= Unclear*

*Score: 1-3= high risk study, 4-5= medium risk study, 6-8= low risk study. Studies in blue are retrospective cohort studies*

|  | **The Newcastle Ottawa scale for quality assessment of a case-control study** |  |
| --- | --- | --- |
|  |  | Author |
|  | Category | Yang et al, 2020 |
| A | Selection |  |
|  | Adequacy of case definition | 🗸 |
|  | Representativeness of cases | 🗸 |
|  | Selection of controls | 🗸 |
|  | Definition of controls (history of disease) | 🗸 |
|  |  |  |
| B | Comparability |  |
|  | Study controls for other variables | 🗸 |
|  |  |  |
| C | Outcome |  |
|  | Assessment of outcome (Independent blind assessment, secured record)? | 🗸 |
|  | Same method of ascertainment of cases/ controls | 🗸 |
|  | Non-response rate (same for both groups)? | 🗸 |
|  |  |  |
|  | Score | 8 |

**U= Unclear*

*Score: 1-3= high risk study, 4-5= medium risk study, 6-8= low risk study*

|  | **The Newcastle Ottawa scale for quality assessment of cross-sectional studies** |  |  |
| --- | --- | --- | --- |
|  |  | Authors |  |
|  | Category | SeyedAlinaghi et al, 2020 | Guo et al, 2020 |
| A | Selection |  |  |
|  | Inclusion and exclusion criteria applied uniformly | 🗸 | 🗸 |
|  | Representativeness of cases | 🗸 | 🗸 |
|  | Account for confounding/modifying variables | 🗸 | 🗸 |
|  | Exposures defined using valid and reliable measures | 🗸 | 🗸 |
|  |  |  |  |
| B | Comparability |  |  |
|  | Study controls for other variables | 🗸 | 🗸 |
|  |  |  |  |
| C | Outcome |  |  |
|  | Outcome assessor blinded to the exposure status | 🗸 | 🗸 |
|  | Outcomes defined using valid and reliable measures | 🗸 | 🗸 |
|  | All pre-specified outcomes reported | 🗸 | 🗸 |
|  |  |  |  |
|  | Score | 8 | 8 |

**U= Unclear*

*Score: 1-3= high risk study, 4-5= medium risk study, 6-8= low risk study*

|  | **The Newcastle Ottawa scale for quality assessment of case series** |  |  |  |  |  |  |  |  |  |
| --- | --- | --- | --- | --- | --- | --- | --- | --- | --- | --- |
|  |  | Authors |  |  |  |  |  |  |  |  |
|  | Category | Altuntas Aydin et al, 2020 | Blanco Jose et al, 2020 | Childs et al, 2020 | Gervasoni et al, 2020 | Gudipati et al, 2020 | Härter et al, 2020 | Hu et al, 2020 | Sasset et al, 2020 | Swaminathan et al, 2020 |
| A | Selection |  |  |  |  |  |  |  |  |  |
|  | Inclusion and exclusion criteria applied uniformly | U | 🗸 | 🗸 | 🗸 | 🗸 | 🗸 | 🗸 | U | 🗸 |
|  | Representativeness of cases | × | 🗸 | 🗸 | 🗸 | × | 🗸 | × | × | × |
|  | Account for confounding/modifying variables | 🗸 | 🗸 | 🗸 | 🗸 | 🗸 | 🗸 | 🗸 | 🗸 | 🗸 |
|  | Cases condition defined using valid and reliable measures | 🗸 | 🗸 | 🗸 | 🗸 | 🗸 | 🗸 | 🗸 | 🗸 | 🗸 |
|  |  |  |  |  |  |  |  |  |  |  |
| B | Comparability |  |  |  |  |  |  |  |  |  |
|  | Compare cases to similar studies | × | × | 🗸 | 🗸 | 🗸 | 🗸 | 🗸 | × | 🗸 |
|  |  |  |  |  |  |  |  |  |  |  |
| C | Outcome |  |  |  |  |  |  |  |  |  |
|  | Adequacy of follow-up of all cases | 🗸 | 🗸 | 🗸 | 🗸 | 🗸 | 🗸 | 🗸 | 🗸 | 🗸 |
|  | Outcomes defined using valid and reliable measures | 🗸 | 🗸 | 🗸 | 🗸 | 🗸 | 🗸 | 🗸 | 🗸 | 🗸 |
|  | Outcome assessor blinded to the exposure status | U | U | U | 🗸 | U | 🗸 | U | U | U |
|  |  |  |  |  |  |  |  |  |  |  |
|  | Score | 4 | 6 | 7 | 8 | 6 | 8 | 6 | 4 | 6 |

**U= Unclear*

*Score: 1-3= high risk study, 4-5= medium risk study, 6-8= low risk study*

|  | **The Newcastle Ottawa scale for quality assessment of a case report study** |  |
| --- | --- | --- |
|  |  | Author |
|  | Category | Tian et al, 2020 |
| A | Selection |  |
|  | Inclusion and exclusion criteria applied uniformly | 🗸 |
|  | Patient’s history and characteristics clearly described | 🗸 |
|  | Account for confounding/modifying variables | 🗸 |
|  | Exposures defined using valid and reliable measures | 🗸 |
|  |  |  |
| B | Comparability |  |
|  | Compare case to similar studies | 🗸 |
|  |  |  |
| C | Outcome |  |
|  | Outcomes clearly described | 🗸 |
|  | Outcomes defined using valid and reliable measures | 🗸 |
|  | All pre-specified outcomes reported | × |
|  |  |  |
|  | Score | 7 |

**U= Unclear*

*Score: 1-3= high risk study, 4-5= medium risk study, 6-8= low risk study*
