# Supplementary material for: Clinical outcomes of COVID-19 amongst HIV patients: a systematic literature review
Source: Epidemiol Health. 2021 May 17;43:e2021036. doi: 10.4178/epih.e2021036 (PMC8342867; doi:10.4178/epih.e2021036)
Supplement: Supplementary Material 3. — Funnel plot for the assessment of publication bias following a meta-analysis of proportion. [file epih-43-e2021036-suppl3.docx]

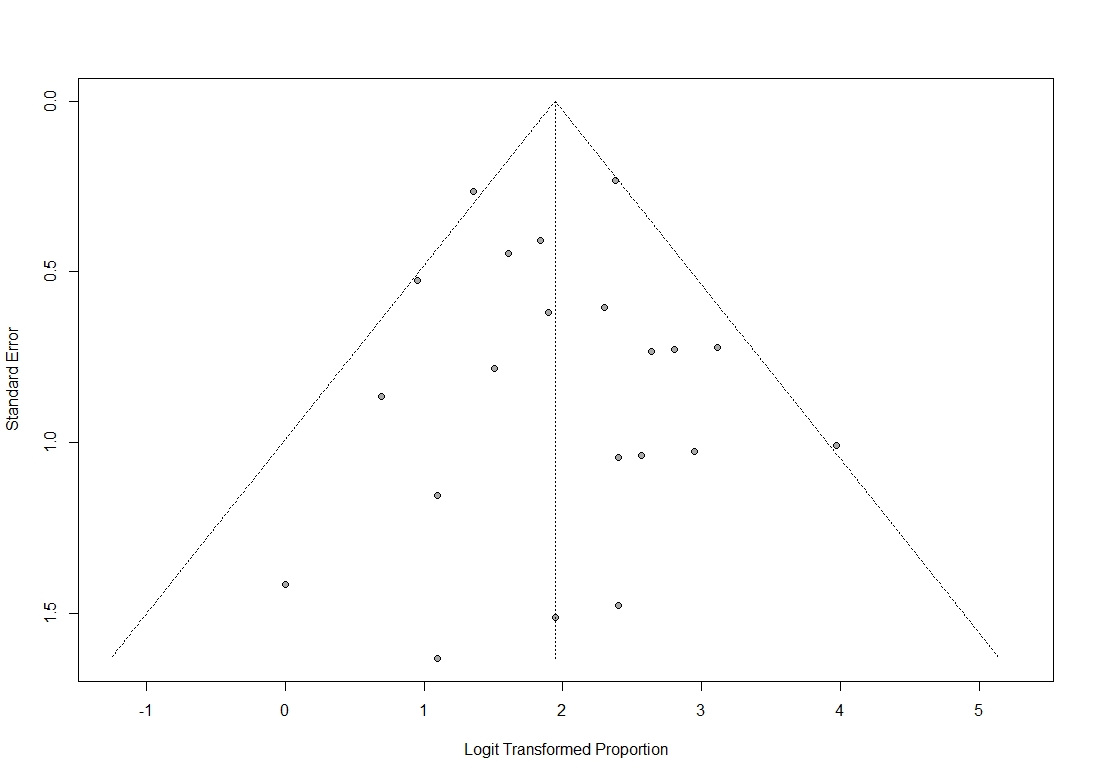


**Supplementary Material 3**. Funnel plot for the assessment of publication bias following a meta-analysis of proportion.
